# Clinical-pharmacological medication reviews for insurants of a German statutory health insurance fund (*Kaufmännische Krankenkasse* (KKH)): a 5-year retrospective analysis (2019–2023)

**DOI:** 10.1007/s00210-026-05741-8

**Published:** 2026-07-22

**Authors:** Johannes Heck, Dirk O. Stichtenoth, Lutz Herbarth, Christoph Schröder, Ruxandra Sabau, Thorben Pape, Sebastian Schröder, Benjamin Krichevsky, Martin Schulze Westhoff, Martin Klietz, Stephan Greten, Patrick-Pascal Strunz, Carsten Schumacher

**Affiliations:** 1 Institute for General Practice and Palliative Care, Hannover Medical School, Carl-Neuberg-Str. 1, 30625 Hannover, Germany; 2 Institute for Clinical Pharmacology, Hannover Medical School, Carl-Neuberg-Str. 1, 30625 Hannover, Germany; 3 Kaufmännische Krankenkasse, Karl-Wiechert-Allee 61, 30625 Hannover, Germany; 4 Department of Respiratory Medicine and Infectious Diseases, Hannover Medical School, Carl-Neuberg-Str. 1, 30625 Hannover, Germany; 5 Department of Psychiatry Social Psychiatry and Psychotherapy, Hannover Medical School, Carl-Neuberg-Str. 1, 30625 Hannover, Germany; 6 Dean’s Office for Academic Career Development, PRACTIS Clinician Scientist Program, Hannover Medical School, Carl-Neuberg-Str. 1, 30625 Hannover, Germany; 7 Interdisciplinary Emergency Department, University Hospital Schleswig-Holstein, Campus Kiel, Arnold-Heller-Str. 3, 24105 Kiel, Germany; 8 Hannover Medical School, Department of Neurology, Hannover Medical School, Carl-Neuberg-Str. 1, 30625 Hannover, Germany; 9 Department of Internal Medicine II, Rheumatology/Clinical Immunology, University Hospital Würzburg, Oberdürrbacher Str. 6, 97080 Würzburg, Germany; 10 Center for Clinical Trials, Hannover Medical School, Feodor-Lynen-Str. 15, 30625 Hannover, Germany

**Keywords:** Clinical pharmacology, Medication review, Statutory health insurance fund, Potentially inappropriate medications, Potentially inappropriate duplicate prescriptions

## Abstract

This study sought to evaluate the characteristics of clinical-pharmacological (CP) medication reviews conducted by the Institute for Clinical Pharmacology of Hannover Medical School for older, multimorbid, polymedicated insurants of the German statutory health insurance fund *Kaufmännische Krankenkasse* (KKH). Special emphasis was put on potential sex-specific differences between female and male insurants, prescriptions of potentially inappropriate medications for older individuals (PIMs), and potentially inappropriate duplicate prescriptions (PIDPs). Medication reviews conducted for KKH insurants between 2019 and 2023 (*n* = 334) were analyzed retrospectively. Clinical trial number is not applicable. The median age of KKH insurants (58.1% female) was 74 years (first quartile (Q1): 68; third quartile (Q3): 80). Insurants displayed a median of 7 diseases (Q1: 5; Q3: 9) and took a median of 13 drugs (Q1: 11; Q3: 16). A median of 4 CP recommendations was issued per medication review (Q1: 3; Q3: 5). The number of CP recommendations was significantly higher for female compared to male insurants (median of 4 (Q1: 3; Q3: 6) vs. median of 3 recommendations (Q1: 2; Q3: 4); *p* < 0.001), while the number of drugs was identical and the number of diagnoses was higher for female insurants. The most frequently prescribed PIMs were tricyclic antidepressants, benzodiazepines, and Z-drugs. At least one PIDP was detected in 16.5% of medication reviews. The sex-specific difference in the number of CP recommendations between female and male insurants may point to an unmet pharmacotherapeutic need in older women. PIM prescriptions and PIDPs were relevant topics of CP recommendations, suggesting possibilities for pharmacotherapeutic optimization in older, multimorbid, polymedicated patients.

## Introduction

Medicines, when chosen wisely and administered appropriately, have the potential to enhance patients’ quality of life and improve health outcomes. Conversely, the inadequate use of medications can lead to ineffective pharmacotherapy and may result in adverse drug reactions (ADRs) (Ernst and Grizzle 2001; Rodríguez-Monguió et al. 2003; Griese-Mammen et al. 2018). The prevalence of medication-related morbidity and mortality presents a substantial public health challenge, and medication errors are increasingly recognized as a cause of injury and preventable harm within healthcare systems globally (Ernst and Grizzle 2001; Rodríguez-Monguió et al. 2003; Griese-Mammen et al. 2018). The World Health Organization (WHO) has addressed this issue through the patient safety initiative Medication Without Harm, underscoring the need for comprehensive strategies to mitigate risks associated with inappropriate pharmacotherapy and medication errors (World Health Organization 2017).

A systematic and comprehensive analysis of a patient’s medication regimen—also termed medication review—is a frequently employed strategy for improving pharmacotherapy (Vogt et al. 2024). The Pharmaceutical Care Network Europe (PCNE) defines a medication review as “a structured evaluation of a patient’s medicines with the aim of optimizing medicines use and improving health outcomes. This entails detecting drug-related problems and recommending interventions” (Griese-Mammen et al. 2018). Medication reviews have been implemented across various healthcare settings and by a range of healthcare professionals (Vogt et al. 2024). However, the scope and depth of medication reviews can vary significantly, reflecting differences in practice standards, clinical contexts, and professional expertise (Vogt et al. 2024). Even though analyzing complex medication regimens is a core competence of clinical pharmacologists (CPs), research on CP-led medication reviews is scarce, in contrast to pharmacist-led medication reviews (Vogt et al. 2024). In addition to CPs and pharmacists, other medical subspecialties and professions are also involved in medication reviews, although their roles vary. While geriatricians typically take over the clinical coordination in their patient population (Sutanto 2025), focusing, for example, on prescribing guideline-recommended medicines (i.e., addressing potential prescribing omissions (PPOs)) and deprescribing potentially inappropriate medications for older individuals (PIMs) (Scott et al. 2026; Wang et al. 2023), general practitioners (GPs) are common reviewers in primary healthcare (Engels et al. 2025). Nurses are recognized as an important link between physicians, pharmacists and patients. Nurses are responsible for recording, verifying, and communicating first-hand information, as well as for the practical implementation of prescriptions and deprescriptions (Mardani et al. 2020). Thereby, nurse-led interventions strive to improve medication reconciliation, patient education, and medication adherence (Mardani et al. 2020; Berardinelli et al. 2024; Persell et al. 2018). However, regardless of the specific division of roles, there is a consensus that an interdisciplinary approach involving physicians, pharmacists, and nurses is beneficial for the quality of medication reviews (Sutanto 2025; Engels et al. 2025; Mardani et al. 2020).

In Germany, several statutory health insurance fund-supported medication management programs exist, in which pharmacists and GPs collaborate, such as the AOK (*Allgemeine Ortskrankenkasse*) PLUS-supported medication management program ARMIN (*Arzneimittelinitiative Sachsen-Thüringen*) (Moecker et al. 2022). However, to the best of our knowledge, the *Kaufmännische Krankenkasse* (KKH) to date is the only German statutory health insurance fund which supports an interdisciplinary medication review collaboration on a national level among physicians, i.e., between CPs and GPs.

Founded in 1890, KKH currently has around 1.5 million insurants, ranking 13th among a total of 72 statutory health insurance funds in Germany with respect to the number of insurants (as of April 1, 2026) (KKH n.d.; 130 Jahre KKH n.d.; Krankenkassen Deutschland n.d.). As a special service, KKH offers its insurants individual telephone-based health coaching to promote a healthy lifestyle and support therapeutic measures (KKH 2024) including the offer of a specialist CP medication review. As part of the cooperation project Safe Use of Medicine (*Arzneimittel sicher anwenden*), which was established in 2012 (Laidig et al. 2018), those expert medication reviews are conducted by the Institute for Clinical Pharmacology of Hannover Medical School. The present study sought to investigate the characteristics of CP (i.e., physician-led) medication reviews that were conducted during a 5-year period (2019–2023). Previous analyses from our institution indicated that PIM prescriptions and potentially inappropriate duplicate prescriptions (PIDPs) constitute important medication-related problems in hospitalized patients (Heck et al. 2022, 2026). Therefore, we aimed to assess whether PIMs and PIDPs also represent clinically relevant pharmcological challenges among older, community-dwelling patients in primary healthcare. In addition, we sought to investigate potential sex-specific differences between female and male insurants, as knowledge about such differences is essential to implement personalized medicine-based approaches in the ambulatory setting. For example, a prospective cohort study of community-dwelling older patients conducted in general practices in the Republic of Ireland showed that female patients were 83% more likely to be affected by ADRs than their male counterparts (odds ratio (OR) 1.83, 95% confidence interval (CI) 1.17–2.85) (Doherty et al. 2023), and female sex was associated with an increased risk for ADR-related hospital admission (Oscanoa et al. 2017; Bellanca et al. 2023). In a study among older community-dwelling United States (US) citizens, female individuals were 69% more likely than men to receive prescriptions of potentially inappropriate sedatives such as alprazolam, lorazepam, or zolpidem (OR 1.69, 95% CI 1.32–2.16) (Extavour and Perri 2018). With its particular focus on PIMs, PIDPs, and potential sex-specific differences, the present study succeeds a previous investigation by Laidig and colleagues, who analyzed potential prescribing errors detected during medication reviews for KKH insurants over a decade ago (2012–2013) (Laidig et al. 2018).

## Methods

### Ethics approval

This study was approved by the Ethics Committee of Hannover Medical School (ethics vote no. 11346_BO_K_2024) and adheres to the Declaration of Helsinki (1964) and its later amendments (current version dating from October 2024). Clinical trial number is not applicable.

### Modus operandi of clinical-pharmacological medication reviews

The design of the project Safe Use of Medicine was described in detail by Laidig et al. (2018). In brief, KKH devised inclusion and exclusion criteria and asked insurants fulfilling the inclusion criteria to participate in the project. Insurants taking 5 to 11 drugs concomitantly (at a cross-sectional moment) and displaying a risk of hospitalization of over 50% (according to an internal algorithm described by Boldt (2015) were asked to participate (Laidig et al. 2018). Insurants taking > 12 drugs were invited to participate irrespective of their risk of hospitalization. Exclusion criteria were presence of oncological disease, need for renal replacement therapy, current hospitalization, or residence in a nursing home (Laidig et al. 2018). Insurants interested in participation were contacted by trained KKH health coaches for a telephone interview after having provided written informed consent. Information provided by insurants during the telephone interview (e.g., information about self-medication) was joined with official KKH account data comprising drug prescription and medical diagnosis information submitted to KKH by physicians and pharmacies for remuneration purposes (Laidig et al. 2018). Medical diagnoses were grouped according to the International Statistical Classification of Diseases and Related Health Problems, 10th revision (ICD-10). Subsequently, all necessary data were compiled, summarized, and made available to the Institute for Clinical Pharmacology in pseudonymized form through a KKH-operated exchange server. Of note, the pseudonymization key remained entirely with KKH; hence, the Institute for Clinical Pharmacology analyzed all data anonymously.

The Institute for Clinical Pharmacology is headed by a physician, and the majority of the operative staff are also physicians, the sole exception being one clinical pharmacist with specialty training in drug information. The modus operandi for medication reviews is laid down in a standard operating procedure (SOP). Briefly, current medication regimens of KKH insurants were checked for correct indications, potential contraindications, correct dosages, clinically relevant potential drug–drug interactions (DDIs), PIM prescriptions, PIDPs, and PPOs, taking into account current medical guidelines, summary of product characteristics, medical databases (e.g., PubMed), textbooks of (clinical) pharmacology (e.g., Goodman & Gilman’s The Pharmacological Basis of Therapeutics), and drug information databases (e.g., AiD*Klinik* (Dosing GmbH, Heidelberg, Germany)). Of note, AiD*Klinik* features an embedded drug interaction program, which was used to determine clinically relevant DDIs in conjunction with clinical judgment. Medication reviews were performed at a single timepoint in the sense of a cross-sectional analysis by residents in CP or by the clinical pharmacist. For each medication review, written recommendations were drafted, discussed with, adjusted, and approved by a senior physician (dual control principle). Hence, while a physician or a pharmacist drafted the preliminary recommendations, it was always a senior physician who was in charge of the final approval of the medication reviews. The finalized recommendations were submitted to KKH through the exchange server mentioned above. All medication reviews were saved locally in anonymized form at the Institute for Clinical Pharmacology in a Microsoft Excel spreadsheet (Redmond, WA, USA) for internal documentation.

The CP recommendations were sent to the insurants’ treating GPs by KKH in de-pseudonymized form, so that GPs were able to discuss the CP recommendations with their patients. GPs subsequently had the possibility to ask for a telephone consultation with the Institute for Clinical Pharmacology to discuss the recommendations on a medical professional level. Importantly, GPs only mentioned the patients’ pseudonyms (i.e., numerical codes) when speaking to the Institute for Clinical Pharmacology. Thus, it was safeguarded that the patients’ true identities remained unknown to the Institute for Clinical Pharmacology and that anonymity was maintained. The points of discussion of those telephone calls between GPs and CPs were summarized by the Institute for Clinical Pharmacology in written form (letter), and concluding remarks (e.g., adjusted final recommendations) were sent to the treating GPs through KKH.

### Data acquisition

For the purpose of this study, all medication reviews conducted for KKH insurants by the Institute for Clinical Pharmacology of Hannover Medical School between 2019 and 2023 were analyzed. For each medication review, the following parameters were assessed: age and sex of the insurant; medical diagnoses; number of drugs taken according to the insurant’s current medication plan (cross-sectional analysis at a single timepoint; prescription medicines and over-the-counter drugs, as well as scheduled drugs and drugs used on an on-demand basis were considered; each pharmacological agent was counted individually, e.g., a combination product containing levodopa and benserazide was counted as two drugs); number of CP recommendations; recommendation categories (indication and contraindication; validation of unclear indications; validation of unclear dosages; application frequency and time of administration; dosage adjustment (e.g., due to renal insufficiency); ADR; drug discontinuation; pharmacodynamic interaction; pharmacokinetic interaction; PIM (according to the German PRISCUS list (Holt et al. 2010; Mann et al. 2023); PIDP (according to the classification by Heck et al. (Heck et al. 2021); grade-1 PIDP: prescription of “two drugs with overlapping or comparable pharmacodynamics” (Heck et al. 2021); grade-2 PIDP: prescription of “two drugs of the same therapeutic class (that is, targeting the same molecular structure)” (Heck et al. 2021); grade-3 PIDP: prescription of “two times the same drug (exceeding the recommended maximum daily dose)” (Heck et al. 2021); therapeutic drug monitoring (TDM); monitoring of pharmacotherapy (other than TDM; e.g., electrolytes, ECG); miscellaneous).

### Statistical analysis

Descriptive statistical techniques were used to summarize the data. For categorical variables, absolute and relative frequencies are presented. Quantitative variables are depicted as medians with first (Q1) and third quartiles (Q3) as the data were not normally distributed. Differences between female and male insurants were analyzed with the Mann–Whitney *U* test. To explore the relationship between the number of drugs taken by insurants and the number of CP recommendations made per medication review, a linear regression analysis was performed, and Pearson’s coefficient of correlation (*R*) and the coeffient of determination (*R*^2^) were calculated. The equation of the regression line was determined as *Y* = *m* × *X* + *c* (*Y*: dependent variable (here: number of CP recommendations); *m*: slope (regression coefficient); *X*: independent variable (here: number of drugs taken); *c*: y-intersect) (Schneider et al. 2010).

*p*-values < 0.05 were considered statistically significant, without adjustment for multiple testing. All statistical analyses were conducted with IBM SPSS Statistics for Windows, version 29 (Armonk, NY, USA).

## Results

### Characteristics of insurants

Between 2019 and 2023, the Institute for Clinical Pharmacology of Hannover Medical School conducted medication reviews for 334 KKH insurants (58.1% female; Table 1). Insurants were between 42 and 85 years old, with a median age of 74 years (Q1: 68; Q3: 80). Female insurants were significantly older than their male counterparts (median 75 years (Q1: 69; Q3: 81) vs. median 72 years (Q1: 67; Q3: 77); *p* = 0.003), but there were no sex-specific differences with respect to blood pressure, renal function, or body mass index (BMI) (Table 2).

**Table 1:** Characteristics of insurants of the German statutory health insurance fund *Kaufmännische Krankenkasse* for whom clinical-pharmacological medication reviews were conducted by the Institute for Clinical Pharmacology of Hannover Medical School between 2019 and 2023 (*n* = 334)

Variable	*n*	%
Sex		
Female	194	58.1
Male	140	41.9
Diseases		
Arterial hypertension	298	89.2
Type 2 diabetes mellitus	214	64.1
Coronary heart disease	142	42.5
Chronic heart failure	106	31.7
Atrial fibrillation	95	28.4
Hypothyroidism	79	23.7
Bronchial asthma	66	19.8
Chronic obstructive pulmonary disease	60	18.0
Status post stroke	42	12.6
Depression	41	12.3
Cataract	32	9.6
Gout	24	7.2

**Table 2:** Comparison of female and male insurants of the German statutory health insurance fund *Kaufmännische Krankenkasse* for whom clinical-pharmacological medication reviews were conducted by the Institute for Clinical Pharmacology of Hannover Medical School between 2019 and 2023

Category	All insurants	Female insurants	Male insurants	*p*-value
	(*n* = 334)	(*n* = 194; 58.1%)	(*n* = 140; 41.9%)	
Age (years)—median (Q1; Q3)	74 (68; 80)	75 (69; 81)	72 (67; 77)	**0.003**
Systolic blood pressure (mmHg)^§^—median (Q1; Q3)	130 (120; 140)	131 (120; 140)	130 (120; 140))	0.417
Diastolic blood pressure (mmHg)^$^—median (Q1; Q3)	79 (70; 80)	79 (70; 82)	80 (70; 80)	0.770
Estimated glomerular filtration rate (ml/min/1.73 m^2^)^&^—median (Q1; Q3)	62 (51; 81)	61 (49; 82)	68 (53; 77)	0.338
Body mass index (kg/m^2^)^#^—median (Q1; Q3)	29.4 (25.5; 34.0)	29.1 (25.2; 33.7)	29.4 (26.4; 34.3)	0.241
Number of diseases—median (Q1; Q3)	7 (5; 9)	7 (6; 9)	7 (5; 9)	**0.030**
Number of drugs taken—median (Q1; Q3)	13 (11; 16)	13 (11; 16)	13 (11; 16)	0.513
Number of clinical-pharmacological recommendations—median (Q1; Q3)	4 (3; 5)	4 (3; 6)	3 (2; 4)	** < 0.001**

*p*-values < 0.05 were considered statistically significant and are highlighted in bold

*Q1* first quartile, *Q3* third quartile

^§^Values refer to 320 insurants (186 females, 134 males)

^$^Values refer to 319 insurants (185 females, 134 males)

^&^Based on the CKD-EPI (Chronic Kidney Disease Epidemiology Collaboration) equation. Values refer to 291 insurants (167 females, 124 males)

^#^Values refer to 332 insurants (193 females, 139 males)

Insurants displayed between 2 and 21 diseases, with a median of 7 diseases (Q1: 5; Q3: 9), and took between 5 and 34 drugs, with a median of 13 drugs (Q1: 11; Q3: 16). The three most prevalent diseases in the study population were arterial hypertension, type 2 diabetes mellitus, and coronary heart disease, affecting 89.2%, 64.1%, and 42.5% of insurants, respectively. Almost half of the study population (45.5%) were obese (according to the definition by the WHO, that is, BMI ≥ 30 kg/m^2 ^(World Health Organization 2024). The number of diseases was slightly higher for female compared to male insurants despite an identical median (median of 7 diseases (Q1: 6; Q3: 9) vs. median of 7 diseases (Q1: 5; Q3: 9);*p* = 0.030), while the number of drugs did not differ between female and male insurants.

### Clinical-pharmacological recommendations

The number of CP recommendations issued per medication review varied between 0 and 11, with a median of 4 recommendations (Q1: 3; Q3: 5; Table 1). The number of CP recommendations was significantly higher for female compared to male insurants (median of 4 (Q1: 3; Q3: 6) vs. median of 3 recommendations (Q1: 2; Q3: 4); *p* < 0.001).

The three most frequent recommendation categories in CP medication reviews were validation of unclear indications, indications and contraindications, and ADRs, being detected in 69.5% (232/334), 69.2% (231/334), and 46.4% (155/334) of all medication reviews, respectively (assignment of multiple recommendation categories per medication review was possible). Clinical examples for the different recommendation categories are showcased in Table 3.

**Table 3:** Absolute and relative frequencies of recommendation categories in clinical-pharmacological medication reviews conducted for insurants of the German statutory health insurance fund *Kaufmännische Krankenkasse* by the Institute for Clinical Pharmacology of Hannover Medical School between 2019 and 2023 (*n* = 334). Assignment of multiple recommendation categories per medication review was possible

Recommendation category	*n*	%	Example(s)
Validation of unclear indications	232	69.5	The indication for pantoprazole is not clear from the patient’s list of diagnoses
Indication and contraindication	231	69.2	In the case of coronary heart disease, there is a clear indication for a statin. Is there an intolerance or contraindication to statins or are there other reasons for not prescribing them to the patient?
Adverse drug reaction	155	46.4	Mirtazapine can cause weight gain as an adverse drug reaction. In view of the patient’s existing adipositas, we recommend reevaluating the prescription of mirtazapine
Application frequency and time of administration	132	39.5	Amlodipine: due to the pharmacokinetics (plasma half-life of over 35 h), once-daily administration (of the complete daily dose) is usually sufficient Due to improved efficacy, we recommend taking simvastatin in the evening instead of in the morning
Dosage adjustment (e.g., due to renal insufficiency)	129	38.6	In patients with moderately impaired renal function (eGFR between 30 and 60 ml/min), a single dose of 0.2 mg moxonidine and a daily dose of 0.4 mg moxonidine should not be exceeded. In patients with severe renal insufficiency (eGFR < 30 ml/min), the maximum daily dose of moxonidine is 0.3 mg
Pharmacodynamic interaction	136	40.7	Ibuprofen impedes the therapeutically desired antiaggregatory effect of acetylsalicylic acid. It should be taken 8 h before or 2 h after acetylsalicylic acid The combination of an NSAID (here: diclofenac), an ACE inhibitor/AT_1_ receptor antagonist (here: ramipril) and a diuretic (here: hydrochlorothiazide) can lead to a decrease in eGFR or to acute renal failure (so-called triple whammy)
Pharmacokinetic interaction	87	26.0	The simultaneous use of sacubitril–valsartan and atorvastatin can lead to increased exposure to atorvastatin. The reason for this is an inhibition of the transport proteins OATP1B1 and OATP1B3 by sacubitril, which mediate the uptake of atorvastatin in hepatocytes. This increases the risk of myopathy/rhabdomyolysis. High doses of atorvastatin should be avoided in combination with sacubitril–valsartan and the patient should be made aware of possible signs of myopathy/rhabdomyolysis The polyvalent cations (Fe^2+^) contained in ferro sanol® duodenal can form complexes with levothyroxine and reduce its effectiveness
Potentially inappropriate duplicate prescription	55	16.5	The co-prescription of amlodipine and lercanidipine constitutes a potentially inappropriate duplicate prescription
Monitoring of pharmacotherapy (other than TDM): electrolytes, ECG, etc	52	15.6	We recommend regular checks of the potassium level during combined use of valsartan and spironolactone The combined administration of amitriptyline, melperone, and ranolazine can lead to a prolongation of the QT_c_ interval and increase the risk of torsade de pointes. If this combination is unavoidable, we recommend regular ECG monitoring
Drug discontinuation	49	14.7	Self-medication with homeopathic remedies: In the absence of proof of efficacy and in the case of polymedication, we recommend avoiding these preparations in order to reduce the number of medicines
Potentially inappropriate medication for older individuals	47	14.1	Due to an altered receptor affinity, benzodiazepines (here: diazepam) may lead to paradoxical states of agitation in older individuals. They can also display a significantly longer half-life and lead to ataxia, sedation and dependence symptoms. If possible, this drug class should be avoided in advanded age
Validation of unclear dosages	6	1.8	The dosage of cholecalciferol is not clearly stated in your patient’s medication plan. We recommend checking this, as there is a risk of vitamin D intoxication in the event of a cholecalciferol overdose
Therapeutic drug monitoring	4	1.2	Digitoxin is a drug with a narrow therapeutic range. We recommend therapeutic drug monitoring
Miscellaneous	95	28.4	Your patient’s extensive analgesic medication indicates difficult pain management. We recommend consulting a pain specialist

*ECG* electrocardiography, *eGFR* estimated glomerular filtration rate, *NSAID* non-steroidal anti-inflammatory drug, *OATP* Organic Anion Transporting Polypeptide, *QT*_*c*_ corrected QT, *TDM* therapeutic drug monitoring

### Relationship between the number of drugs and the number of clinical-pharmacological recommendations

**Fig. 1:**
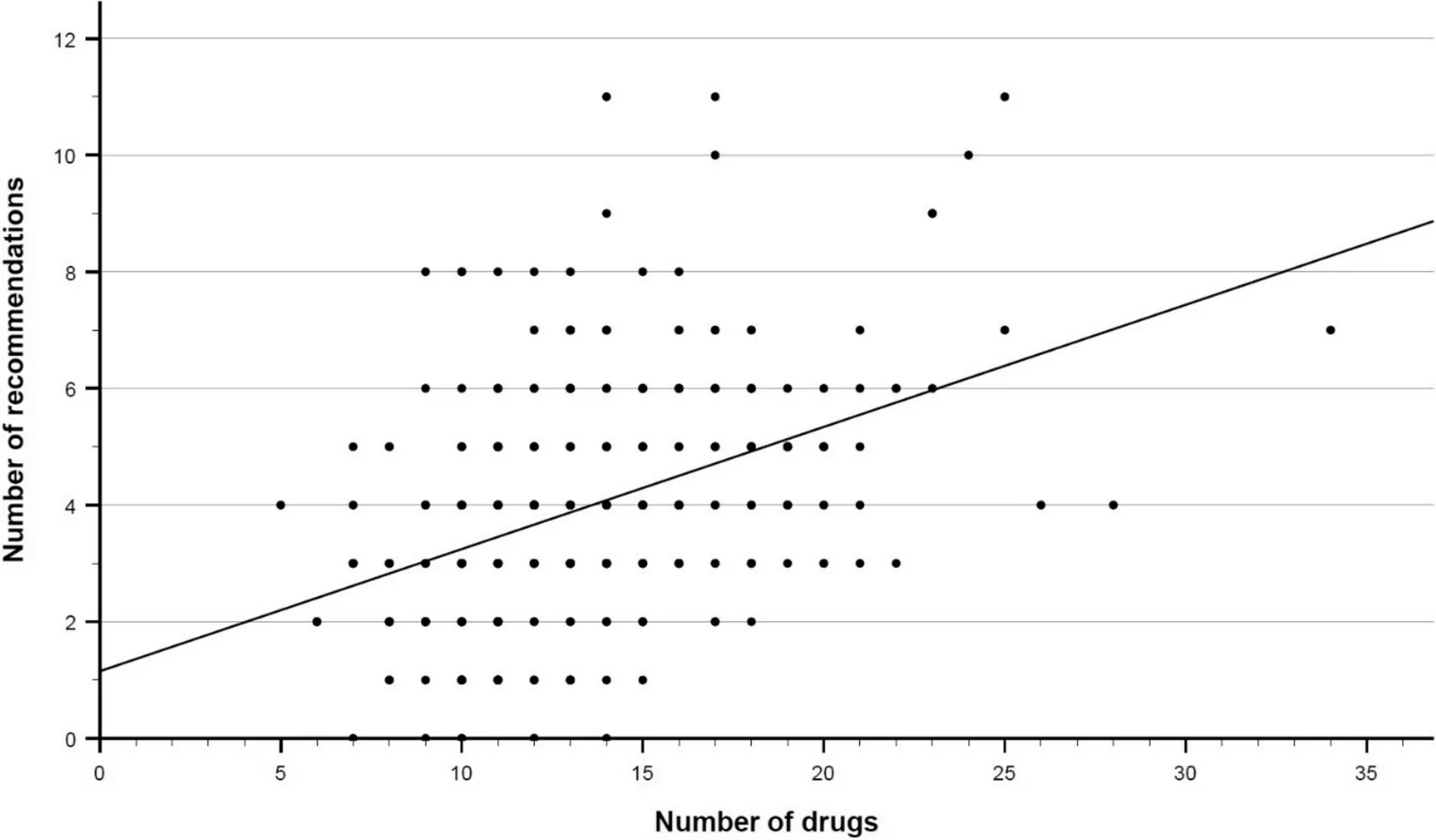
Number of clinical-pharmacological recommendations per medication review in relation to number of drugs taken by insurants of the German statutory health insurance fund *Kaufmännische Krankenkasse*. The equation of the regression line is *Y* = 0.21 × *X* + 1.15, where *Y* is the number of clinical-pharmacological recommendations per medication review and *X* is the number of drugs taken. Pearson’s correlation coefficient (*R*) and the coefficient of determination (*R*^2^) were determined as 0.423 and 0.179, respectively

*R* and *R*^2^ were calculated as 0.423 and 0.179, respectively, suggesting a weak positive linear relationship between the number of drugs taken by insurants and the number of CP recommendations (Fig. 1). The equation of the regression line was determined as *Y* = 0.21 × *X* + 1.15 (*p*-value of the model < 0.001).

### Potentially inappropriate medications for older individuals

PIM prescriptions leading to CP recommendations were detected in 14.1% (47/334) of all medication analyses, comprising a total of 54 individual PIM prescriptions. The most frequently prescribed PIMs that triggered CP recommendations were amitriptyline, bromazepam, doxazosin, opipramol, trimipramine, zolpidem, and zopiclone, accounting for 7.4% (4/54) of PIM prescriptions each (Table 4). On an aggregated level, tricyclic antidepressants, benzodiazepines and Z-drugs constituted the most frequently prescribed PIM classes, accounting for 27.8% (15/54), 25.9% (14/54), and 14.8% (8/54) of PIM prescriptions.

**Table 4:** Prescriptions of potentially inappropriate medications for older individuals (PIMs) leading to clinical-pharmacological recommendations. In total, 54 PIM prescriptions were detected in the study population

Potentially inappropriate medication for older individuals	*n*	%
Amitriptyline	4	7.4
Bromazepam	4	7.4
Doxazosin	4	7.4
Opipramol	4	7.4
Trimipramine	4	7.4
Zolpidem	4	7.4
Zopiclone	4	7.4
Diazepam	3	5.6
Clonidine	2	3.7
Doxepin	2	3.7
Lorazepam	2	3.7
Naftidrofuryl	2	3.7
Oxazepam	2	3.7
Doxylamine	1	1.9
Fluphenazine	1	1.9
Flurazepam	1	1.9
Ketotifen	1	1.9
Medazepam	1	1.9
Metoclopramide	1	1.9
Nitrofurantoin	1	1.9
Nortriptyline	1	1.9
Pentoxifylline	1	1.9
Piracetam	1	1.9
Propiverine	1	1.9
Solifenacin	1	1.9
Temazepam	1	1.9

### Potentially inappropriate duplicate prescriptions

At least one PIDP was detected in 16.5% (55/334) of all medication analyses (at least one grade-1 PIDP: 4.5% (15/334); at least one grade-2 PIDP: 10.2% (34/334); at least one grade-3 PIDP: 3.9% (13/334)). Overall, 68 PIDPs were present in the study population. The majority of PIDPs were of grade 2 (52.9%; 36/68) (Table 5). Sedatives (31.3% (5/16)), inhalatives (30.6% (11/36)), and anti-Parkinson agents (31.3% (5/16)) were most frequently responsible for grade-1, grade-2, and grade-3 PIDPs, respectively.

**Table 5:** Absolute and relative frequencies of potentially inappropriate duplicate prescriptions (PIDPs). In total, 68 PIDPs were detected in the study population

Potentially inappropriate duplicate prescriptions	*n*	%
Grade-1 PIDPs	**16**	**100**
Sedatives	**5**	**31.3**
Lorazepam + zolpidem	3	18.8
Bromazepam + zopiclone	1	6.3
Clonazepam + zolpidem	1	6.3
Antidepressants	**3**	**18.8**
Amitriptyline + duloxetine	1	6.3
Amitriptyline + opipramol	1	6.3
Fluoxetine + opipramol	1	6.3
Platelet aggregation inhibitors	**3**	**18.8**
Clopidogrel + low-dose acetylsalicylic acid^§^	3	18.8
RAAS inhibitors	**3**	**18.8**
Candesartan + ramipril	3	18.8
Analgesics	**2**	**12.5**
Diclofenac + metamizole	1	6.3
Etoricoxib + ibuprofen	1	6.3
Grade-2 PIDPs	**36**	**100**
Inhalatives^$^	**11**	**30.6**
Fenoterol + formoterol	3	8.3
Formoterol + salbutamol	2	5.6
Aclidinium + ipratropium	1	2.8
Budesonide + beclometasone	1	2.8
Fenoterol + indacaterol	1	2.8
Fenoterol + salbutamol	1	2.8
Formoterol + reproterol	1	2.8
Ipratropium + tiotropium	1	2.8
Calcium channel blockers	**6**	**16.7**
Amlodipine + lercanidipine	3	8.3
Lercanidipine + nifedipine	2	5.6
Amlodipine + nifedipine	1	2.8
Analgesics	**5**	**13.9**
Codeine + tilidine	1	2.8
Diclofenac + indometacin	1	2.8
Fentanyl + morphine	1	2.8
Oxycodone + tapentadol	1	2.8
Tilidine + tramadol	1	2.8
Diuretics	**5**	**13.9**
Furosemide + torasemide	2	5.6
Chlortalidone + hydrochlorothiazide	1	2.8
Chlortalidone + xipamide	1	2.8
Furosemide + piretanide	1	2.8
Sedatives	**3**	**8.3**
Diazepam + flunitrazepam	1	2.8
Diazepam + lorazepam	1	2.8
Diazepam + medazepam	1	2.8
Miscellaneous	**6**	**16.7**
Acetazolamide + dorzolamide	1	2.8
Alfuzosin + tamsulosin	1	2.8
Desloratadine + fexofenadine	1	2.8
Eplerenone + spironolactone	1	2.8
Ropinirole + rotigotine	1	2.8
Solifenacin + trospium chloride	1	2.8
Grade-3 PIDPs	**16**	**100**
Anti-Parkinson agents	**5**	**31.3**
Benserazide + benserazide	2	12.5
Levodopa + levodopa	2	12.5
Ropinirole + ropinirole	1	6.3
Analgesics	**4**	**25.0**
Metamizole + metamizole	2	12.5
Ibuprofen + ibuprofen	1	6.3
Paracetamol + paracetamol	1	6.3
Inhalatives	**3**	**18.8**
Formoterol + formoterol	1	6.3
Olodaterol + olodaterol	1	6.3
Tiotropium + tiotropium	1	6.3
Miscellaneous	**4**	**25.0**
Cholecalciferol + cholecalciferol	2	12.5
Low-dose acetylsalicylic acid + low-dose acetylsalicylic acid	1	6.3
Ramipril + ramipril	1	6.3

*PIDP* potentially inappropriate duplicate prescription, *RAAS* renin–angiotensin–aldosterone system

^§^The combination of clopidogrel and low-dose acetylsalicylic acid was only considered potentially inappropriate if the guideline-recommended treatment duration of dual antiplatelet therapy was exceeded

^$^Combinations of short- and long-acting inhalatives were only considered potentially inappropriate if both drugs were applied at the same time point on a regular basis

### Discussion of clinical-pharmacological recommendations with treating general practitioners

CP recommendations were discussed via telephone consultations with the insurants’ treating GPs in 10.8% (36/334) of cases (upon GPs’ request).

## Discussion

### Summary of findings and comparison with other studies

In this study, we analyzed 334 medication reviews conducted for older, multimorbid, and polymedicated insurants of a large German statutory health insurance fund (*Kaufmännische Krankenkasse*(KKH)) by the Institute for Clinical Pharmacology of Hannover Medical School between 2019 and 2023 as part of the project Safe Use of Medicine. On average, insurants (median age 74 years; 58% female) displayed 7 diseases and took 13 drugs. Per medication review, a median of 4 CP recommendations for pharmacotherapeutic optimization was issued. The scope of the present investigation is comparable with a previous study by Laidig et al. conducted over a decade ago (from 2012 to 2013), which analyzed 400 medication reviews (Laidig et al. 2018). Compared to the Laidig et al. study, the proportion of female KKH insurants increased from 48% (Laidig et al. 2018)to 58% in the present investigation. While KKH insurants were slightly older than in the Laidig et al. study (74 vs. 70 years on average (Laidig et al. 2018), the most frequent diagnosis among KKH insurants remained unchanged (arterial hypertension). Also, the second to fifth most frequent diseases were identical in both studies (type 2 diabetes mellitus, coronary heart disease, chronic heart failure, and atrial fibrillation), even though their order slightly differed (Laidig et al. 2018). Notably, there was an increase in the proportion of patients with type 2 diabetes mellitus from 49% (Laidig et al. 2018) to 64%. The proportion of obese insurants (i.e., BMI ≥ 30 kg/m^2 ^(World Health Organization 2024), by contrast, remained identical (46% in both studies) (Laidig et al. 2018).

Of note, in the present investigation, female insurants were significantly older than male insurants (75 vs. 72 years), which may reflect the higher life expectancy of women compared to men in Germany (83 vs. 78 years according to the period life Table 2021/2023 of the Federal Statistical Office (Statistisches Bundesamt (Federal Statistical Office) 2024). The intriguing finding that female insurants—while displaying a higher number of diseases and taking the same number of drugs—received one CP recommendation more than male insurants on average may point to an unmet pharmacotherapeutic need in older women. While PPOs might account for this observed sex-specific difference, this hypothesis remains speculative and needs to be investigated in future studies.

We detected a weak positive linear relationship between the number of drugs taken by KKH insurants and the number of CP recommendations issued per medication review. The coefficient of determination (*R*^2^) was determined as 0.179, indicating that (only) 17.9% of the variance in the number of CP recommendations was due to the number of drugs taken (Schneider et al. 2010). This finding may potentially suggest that polypharmacy is not per se detrimental to patients, but that one must strictly differentiate between appropriate and inappropriate polypharmacy, taking patient-specific factors such as clinical state (e.g., frailty), medical diagnoses, treatment goals, potential DDIs, drug tolerability, as well as patient preferences into account (Haefeli and Meid 2018). If all cases of polypharmacy in the present investigation had been inappropriate, we would have expected a much stronger correlation between the number of drugs taken and the number of CP recommendations. Of note, the same rationale applies to prescribing cascades, as outlined by Dreischulte and colleagues (Dreischulte et al. 2022). Recommendations addressing prescribing cascades were not considered as a separate recommendation category in the present study, but might be worth investigating in further detail in follow-up studies. The regression coefficient of 0.21 in our regression equation (Fig.1) reflects that over the range of 5 to 34 drugs one additional CP recommendation will arise during the medication review for every five additionally prescribed drugs (Schneider et al. 2010).

### Reflections on potentially inappropriate medications for older individuals

Studies by Stuhec and co-workers showed that clinical pharmacist-led medication reviews—in cooperation with GPs—in the primary care setting in Slovenia led to reductions in the number of patients’ medications, number of potential DDIs, and number of PIM prescriptions (Stuhec 2021; Stuhec et al. 2019, 2021). Analogous to our study, Stuhec et al. used the PRISCUS list (Holt et al. 2010)to detect PIMs (Stuhec et al. 2019, 2021). There was a considerable overlap in the most frequently prescribed PIMs between the Stuhec et al. studies and our analysis, with a predominance of benzodiazepines (e.g., bromazepam, diazepam) and Z-drugs (e.g., zolpidem, zopiclone) (Stuhec et al. 2019, 2021), which should generally be avoided in older individuals due to the risk of sedation, cognitive impairment, and risk of falling (Holt et al. 2010). Also, paradoxical reactions such as irritability and agitation have been described for these drugs when used in older patients (Holt et al. 2010). Between 29.2% and 48.9% of recommendations made by clinical pharmacists were accepted by the treating GPs in the Stuhec et al. studies (Stuhec et al. 2019, 2021). In a study by Koyama et al., which analyzed national health insurance claims data in Japan from 2010 to 2015, physicians’ acceptance rates of community pharmacist-conducted medication reviews were even higher, ranging between 80.6% and 94.8% (Koyama et al. 2018). In the future, it will be exciting to investigate GPs’ acceptance of CP (i.e., physician-led) medication reviews. It should be mentioned that the proportion of insurants’ treating physicians who requested a telephone consultation with the Institute for Clinical Pharmacology to discuss recommendations decreased between the Laidig et al. study (Laidig et al. 2018)and our investigation from 13.5% to 10.8%, but the reason for this remains elusive. In the Laidig et al. study, physician feedback to CP medication reviews was mostly neutral (49%) or positive (37%) (Laidig et al. 2018).

### Reflections on potentially inappropriate duplicate prescriptions

It is remarkable that grade-2 PIDPs were more frequent than grade-1 and grade-3 PIDPs in the present study, in contrast to previous investigations in the emergency department (ED) (Heck et al. 2023)and geriatric psychiatry (Schulze Westhoff et al. 2023), where grade-1 PIDPs predominated. In a retrospective cohort study by Heck et al. in the ED (internal medicine), non-opioid analgesics were most frequently responsible for grade-1 PIDPs, whereas inhalatives accounted for the majority of grade-2 and grade-3 PIDPs (Heck et al. 2023). In a cross-sectional study by Schulze Westhoff and colleagues in geriatric psychiatry, grade-1 and grade-2 PIDPs were most frequently elicited by sedatives, while analgesics accounted for all grade-3 PIDPs (Schulze Westhoff et al. 2023). In the present investigation, sedatives accounted for the majority of grade-1 PIDPs, in accordance with Schulze Westhoff et al. in geriatric psychiatry (Schulze Westhoff et al. 2023). In line with Heck et al. in the ED (internal medicine) (Heck et al. 2023), inhalatives were most frequently responsible for grade-2 PIDPs in the present analysis. These congruent findings might be owing to the disease profiles of KKH insurants in the present study, which comprised both psychiatric disorders (e.g., depression (12.3% of insurants)) as well as a plethora of internal medicine diagnoses (e.g., arterial hypertension (89.2%), type 2 diabetes mellitus (64.1%), and coronary heart disease (42.5%)). As a novel finding, anti-Parkinson agents were detected as a major driver of grade-3 PIDPs in the present study. Potential overdosage—the hallmark of grade-3 PIDPs (Heck et al. 2021)—of anti-Parkinson agents such as levodopa may cause cardiac arrhythmias, confusion, nausea, and vomiting (Roche Products Limited 2020).

### Strengths and limitations

Based on the classification system for medication reviews set forth by the PCNE, medication reviews conducted at the Institute for Clinical Pharmacology of Hannover Medical School are equivalent to type 3 (i.e., the highest standard, taking into account drug prescription data, patient interviews (by KKH health coaches) as well as clinical data (e.g., blood pressure, renal function, etc.)) (Pharmaceutical Care Network Europe (PCNE) 2013a, b). This can be regarded as a strength of the present study since a recent systematic review by Vogt et al. showed that the majority (67%) of community pharmacist-led medication review studies were only of PCNE type 2a (i.e., taking into account drug prescription data and patient interviews but not clinical data) (Vogt et al. 2024). A randomized controlled trial by Kari and colleagues in Finland suggested that clinical pharmacist-led medication reviews as part of a people-centered care model (PCNE type 3) were both medically and cost-effective in multimorbid community-dwelling older individuals since “the mean total costs were lower and the generated QALYs [quality-adjusted life years] higher in the intervention group than in the usual care group” (Kari et al. 2022). The average age of the participants (81 vs. 74 years), the proportion of female participants (64.6% vs. 58.1%), the average number of drugs taken (12 vs. 13), as well as the number of identified drug-related problems and resulting recommendations (5 vs. 4) were overall comparable between the Kari et al. study (Kari et al. 2022) and our analysis.

The main limitation of our study arises from its retrospective design; hence, it was not possible to investigate prospective outcome measures such as drug-related hospital admissions, health-related quality of life, or pain relief (Beuscart et al. 2018). Notwithstanding, four out of the seven outcome parameters proposed by Beuscart et al. as an international core outcome set for medication review studies in multimorbid older patients with polypharmacy were addressed in the present investigation, either explicitly (PIMs, clinically significant DDIs) or implicitly (drug overuse, drug underuse) (Beuscart et al. 2018). By contrast, the abovementioned community pharmacist-led medication review studies assessed by Vogt et al. only addressed a median of 0.5 of international core outcomes (Vogt et al. 2024; Beuscart et al. 2018). Moreover, PIDPs—which are subsumed in the outcome drug overuse under the term therapeutic duplication by Beuscart et al. (Beuscart et al. 2018)—were investigated as a separate entity and by taking advantage of a much more nuanced classification system (Heck et al. 2021)in the present study. On the other hand, anticholinergic and sedative substances represent important causes of ADRs, an aspect which is not fully covered by the PIDP concept (Heck et al. 2021). To assess the potential clinical impact of anticholinergic and sedative agents, more specific tools such as the anticholinergic burden score for German prescribers (Kiesel et al. 2018)might have provided greater insight, and we suggest that future studies address those topics using a more refined methodology. Also, indication and contraindication could have been analyzed as separate recommendation categories; however, we opted for a joint analysis to facilitate comparability of our results with previous investigations from our institution (Heck et al. 2022, 2026).

In the present study, the PRISCUS list was chosen as a PIM detection tool because it is specifically tailored to the German prescribing context and the German pharmaceutical market (e.g., regarding approval status and availability of drugs) (Holt et al. 2010; Mann 2023). As a potential limitation, the PRISCUS list only provides a checklist-style enumeration of PIMs, while other more sophisticated tools such as Beers criteria (American Geriatrics Society Beers Criteria® Update Expert Panel 2023)or Screening Tool of Older Persons’ Prescriptions (STOPP) and Screening Tool to Alert to Right Treatment (START) (O’Mahony et al. 2023)take additional criteria for identifying PIMs into account (Fujita et al. 2023). Beers criteria, for example, assess medication not only against older age but also consider underlying diseases, potentially hazardous substance combinations, as well as kidney function (American Geriatrics Society Beers Criteria® Update Expert Panel 2023). STOPP/START criteria enhance the concept of withdrawing inadequate medications by means of adding beneficial pharmacotherapy through detection of PPOs as a counterpart to PIMs (O’Mahony et al. 2023). STOPP/START criteria also consider concomitant diseases, being organized according to physiological categories, such as the cardiovascular or respiratory system (O’Mahony et al. 2023). The STOPP criteria additionally refer to parameters like risk of fall or opioid therapy (O’Mahony et al. 2023). The median number of 4 recommendations per medication review in our study may appear relatively low, and application of Beers criteria (American Geriatrics Society Beers Criteria® Update Expert Panel 2023) or STOPP/START criteria (O’Mahony et al. 2023) instead of the PRISCUS list might potentially have yielded further and more in-depth recommendations. However, those approaches would have exceeded the concept of a broad-based approach of the present study, which was intended to provide comprehensive analysis in accordance with local standard procedures. Moreover, the mere number of CP recommendations does not necessarily translate into a better prescribing quality by GPs. CP recommendations need to be read, evaluated, and finally put into practice by GPs, a process which can be onerous and time-consuming. Too many CP recommendations could potentially lead to over-alerting and alert fatigue among GPs. Therefore, the Institute for Clinical Pharmacology of Hannover Medical School has adopted a policy of concentrating on the clinically most relevant topics during medication reviews in order to avoid alert fatigue among prescribers.

In line with advice from the literature, an approach for future examinations could be to incorporate a variety of screening tools, which seems increasingly feasible in light of the advancing automation of analyses (Bjerre et al. 2026). In addition to the instruments mentioned above, the analysis of cascade therapies could also be included in such a comprehensive approach. Prescribing cascades occur when medications are used to treat the side effects of other medications rather than the underlying disease itself. This constellation can trigger a chain reaction resulting in an increasing number of secondary complications due to new adverse reactions or interactions (Sutanto 2025).

The term deprescribing was first introduced into the health literature by Michael Woodward in an article published in an Australian hospital pharmacy journal in 2003 (Woodward 2003)and has been described as the “process of withdrawal of an inappropriate medication, supervised by a health care professional with the goal of managing polypharmacy and improving outcomes” by Reeve and colleagues through a systematic review of existing definitions (Reeve et al. 2015). Importantly, deprescribing “is not about denying effective treatment to eligible patients. It is a positive, patient-centered intervention, with inherent uncertainties, and requires shared decision making, informed patient consent, and close monitoring of effects” (Scott et al. 2015). Medication reviews—as performed in our study—have been proposed as an effective measure to counteract the estimated rate of 10% overprescribing of medications in general practice (Doherty et al. 2023). A study from Canada showed that electronic clinical decision support combined with routine medication reviews resulted in a 23.7% increase in deprescribing of PIMs for older adults, leading the authors to conclude “that medication reviews should incorporate deprescribing as part of usual care” (McDonald et al. 2025). Authoritative online resources such as the website deprescribing.org may prove useful in this context (Bellanca et al. 2023; University 2026). In the present investigation, deprescribing was not analyzed as a separate recommendation category but was subsumed in the category “drug discontinuation.” Owing to well-established clinical benefits for both inpatients and outpatients (Reeve et al. 2015; Scott et al. 2015), deprescribing should be evaluated as a separate recommendation category in future studies.

The* p*-values resulting from Mann–Whitney *U* tests (eight tests in total; Table 2) were not adjusted for multiple testing due to the exploratory and hypothesis-generating nature of our study. All results must therefore be interpreted with caution and will need to be corroborated in future studies applying a more robust methodology such as a multivariable regression model.

The cost–benefit ratio has been cited as a major obstacle to implementing interdisciplinary medication management programs (Moecker et al. 2022), potentially limiting the transferability of our results to other settings, especially less affluent healthcare systems. Interestingly, the average number of drugs taken by KKH insurants did not change between the predecessor study by Laidig et al. (Laidig et al. 2018)and our analysis (13 drugs in both studies). However, as outlined above, the mere number of drugs alone is probably insufficient for evaluating the quality of complex medication regimens (Haefeli and Meid 2018; Dreischulte et al. 2022). Of note, we cannot rule out that the results of the study by Laidig and colleagues from 2012/2013 (Laidig et al. 2018) influenced the prescribing behavior of GPs and/or the medication reviewers at the Institute for Clinical Pharmacology in the present investigation, leading to potential bias. In the future, a follow-up study should investigate the effects of CP medication reviews on prospective outcome measures that are both medically (e.g., reduction of ADRs, prevention of ADR-related hospital admissions, increased quality of life) as well as economically relevant (e.g., minimization of drug expenses through reduction of PIDPs).

### Outlook for medication reviews

Pharmacogenetics is an emerging element of personalized medicine and has the potential to improve efficacy and safety of pharmacotherapy (Bellanca et al. 2023; Fujita et al. 2023; Bollinger et al. 2023; O’Shea et al. 2022). While pharmacogenetic testing is already established as an important facet of specialist pharmacotherapeutic consultation services for hospitalized patients (Heck et al. 2022, 2026; Hjemås et al. 2023), it is not yet routinely integrated into the management of ambulatory patients in general practice, despite efforts pointing into this direction (O’Shea et al. 2022; Hayward et al. 2021). For example, a study by Bollinger et al. showed that pairing medication reviews with pharmacogenetic information on 30 genes was beneficial for patients affected by ADRs or therapy failure in primary and secondary care settings in Switzerland (Bollinger et al. 2023). Advantages of pharmacogenetic testing were most pronounced for patients taking drugs for mental or behavioral disorders and immunological, circulatory, and pain-related diseases, as well as for older patients affected by polypharmacy (Bollinger et al. 2023). Future studies should aim at systematically embedding pharmacogenetic information into medication reviews.

Another aspect becoming increasingly important for medication reviews is artificial intelligence (AI). AI tools have been credited with potential capabilities regarding polypharmacy management, such as PIM detection, DDI prediction, enforcing safety, promoting treatment adherence, and risk evaluation regarding medication-related errors (Fujita et al. 2023; Bringhurst et al. 2025). For example, in a 2024 study by Sridharan and Sivaramakrishnan, four advanced large language models (LLMs)—ChatGPT-3.5, Gemini Pro, Claude-Instant, and Llama-2-13b—showed promise in addressing dosing regimen errors, DDIs, TDM, and pharmacogenetic aspects during medication reviews when the investigated (albeit fictitious) case vignettes were straightforward and when precise clinical questions were formulated (Sridharan and Sivaramakrishnan 2024). By contrast, a real-world study from our working group demonstrated that the quality of information of responses generated by ChatGPT-3.5 to complex pharmacotherapeutic queries was substantially inferior and the error rate was significantly higher compared to conventional, human expert-generated responses (Krichevsky et al. 2026). Therefore, to date, we caution against putting unreserved trust into plausible- and authoritative-sounding but potentially erroneous LLM-generated answers to complex medication-related questions. Nonetheless, based on structured coding of PIM criteria, AI could be used for continuous health data screening with a particular focus on PIM detection in the future. This could reduce the need for periodic manual data maintenance and potentially increase the overall efficiency of medication reviews (Bjerre et al. 2026). However, it is crucial to align such tools meticulously with the user requirements as an excess of information and resulting alerts—but also factually wrong information—could seriously undermine the potential for an overall benefit (Abuzour et al. 2026). Subsequent studies should therefore aim to identify the optimal combination of screening tools for AI models that not only offer improvements from a medical and economic perspective, but are also beneficial from the view of the end user.

### Conclusion and relevance for clinical practice

The sex-specific difference in the number of CP recommendations between female and male insurants detected in our study may point to an unmet pharmacotherapeutic need in older women, although further studies are necessary to shed light on the underlying reasons. PIM prescriptions and PIDPs emerged as relevant topics of CP recommendations for GPs, suggesting possibilities for pharmacotherapeutic optimization in older, multimorbid, polymedicated, community-dwelling patients.

## Data Availability

The data that support the findings of this study are available upon reasonable request from the corresponding author.

## Abbreviations

ADR: Adverse drug reaction
AI: Artificial intelligence
AOK: Allgemeine Ortskrankenkasse (General local health insurance fund)
ARMIN: Arzneimittelinitiative Sachsen-Thüringen (Medicines Initiative Saxony–Thuringia)
BMI: Body mass index
CI: Confidence interval
CKD-EPI: Chronic kidney disease epidemiology collaboration
CP: Clinical pharmacology; clinical pharmacologist; clinical-pharmacological
DDI: Drug–drug interaction
eGFR: Estimated glomerular filtration rate
ECG: Electrocardiography
ED: Emergency department
GFR: Glomerular filtration rate
GP: General practitioner
ICD-10: International statistical classification of diseases and related health problems, 10th revision
KKH: *Kaufmännische Krankenkasse*
LLM: Large language model
no.: Number
NSAID: Non-steroidal anti-inflammatory drug
OR: Odds ratio
PCNE: Pharmaceutical care network Europe
PIM: Potentially inappropriate medication for older people
PIDP: Potentially inappropriate duplicate prescription
PPO: Potential prescribing omission
QALY: Quality-adjusted life year
QTc: Corrected QT
SOP: Standard operating procedure
START: Screening tool to alert to right treatment
STOPP: Screening tool of older persons’ prescriptions
TDM: Therapeutic drug monitoring
US: United States
WHO: World Health Organization
